# 3,5-Bis[1-acetyl-5-(4-chloro­phen­yl)-4,5-dihydro-1*H*-pyrazol-3-yl]-2,6-dimethyl­pyridine

**DOI:** 10.1107/S1600536808023921

**Published:** 2008-08-06

**Authors:** Qun Qian, Jun Zhang, Min Zhang, Xiang He, Yi-Ben Xia

**Affiliations:** a Department of Chemistry, College of Science, Shanghai University, Shanghai 200444, People’s Republic of China; b School of Materials Science and Engineering, Shanghai University, Shanghai 200072, People’s Republic of China

## Abstract

The title compound, C_29_H_27_Cl_2_N_5_O_2_, contains a central pyridine ring and two functionalized pyrazoline rings. The pyridine ring and the two attached pyrazoline rings are nearly coplanar, whereas the terminal chloro­phenyl rings are nearly perpendicular to the attached pyrazoline rings [dihedral angles = 86.78 (1) and 77.70 (1)°]. Mol­ecules are linked by weak inter­molecular C—H⋯O hydrogen bonding.

## Related literature

For general background, see: Ahn *et al.* (2004 ▶); Palaska *et al.* (1996 ▶); Yar *et al.* (2006 ▶)
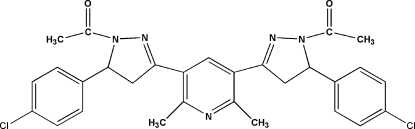


## Experimental

### 

#### Crystal data

C_29_H_27_Cl_2_N_5_O_2_
*M* *_r_* = 548.46
Monoclinic, 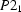

*a* = 12.345 (3) Å
*b* = 9.6763 (19) Å
*c* = 13.268 (3) Å
β = 115.00 (3)°
*V* = 1436.4 (5) Å^3^
*Z* = 2
Mo *K*α radiation
μ = 0.26 mm^−1^
*T* = 296 (2) K
0.30 × 0.20 × 0.20 mm

#### Data collection

Bruker SMART CCD area-detector diffractometer
Absorption correction: multi-scan (*SADABS*; Sheldrick, 1996 ▶) *T*_min_ = 0.926, *T*_max_ = 0.950
7509 measured reflections
3736 independent reflections
2520 reflections with *I* > 2σ(*I*)
*R*_int_ = 0.027

#### Refinement

*R*[*F* ^2^ > 2σ(*F* ^2^)] = 0.036
*wR*(*F* ^2^) = 0.077
*S* = 0.91
3736 reflections
344 parameters
1 restraint
H-atom parameters constrained
Δρ_max_ = 0.14 e Å^−3^
Δρ_min_ = −0.15 e Å^−3^
Absolute structure: Flack (1983 ▶), 1031 Friedel pairs
Flack parameter: 0.06 (6)

### 

Data collection: *SMART* (Bruker, 2000 ▶); cell refinement: *SAINT* (Bruker, 2000 ▶); data reduction: *SAINT*; program(s) used to solve structure: *SHELXTL* (Sheldrick, 2008 ▶); program(s) used to refine structure: *SHELXTL*; molecular graphics: *SHELXTL*; software used to prepare material for publication: *SHELXTL*.

## Supplementary Material

Crystal structure: contains datablocks I, global. DOI: 10.1107/S1600536808023921/xu2444sup1.cif

Structure factors: contains datablocks I. DOI: 10.1107/S1600536808023921/xu2444Isup2.hkl

Additional supplementary materials:  crystallographic information; 3D view; checkCIF report

## Figures and Tables

**Table 1:** Hydrogen-bond geometry (Å, °)

*D*—H⋯*A*	*D*—H	H⋯*A*	*D*⋯*A*	*D*—H⋯*A*
C9—H9*A*⋯O2^i^	0.99	2.59	3.358 (3)	135
C17—H17*A*⋯O2^ii^	0.95	2.50	3.359 (4)	151

Symmetry codes: (i) 
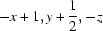
; (ii) 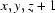.

**Fig. 1.**
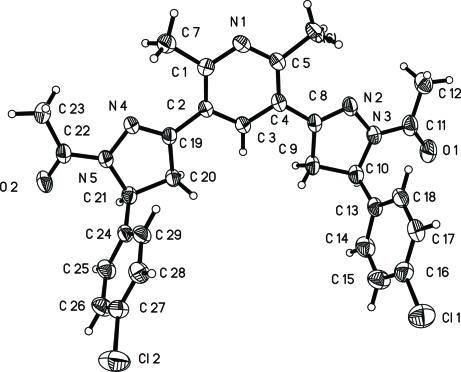
The molecular structure of the title molecule, with the atom-numbering scheme. Displacement ellipsoids are drawn at the 30% probability level.

**Fig. 2.**
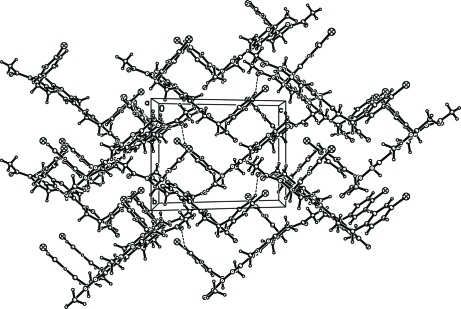
Crystal packing diagram of compound (I), Hydrogen bonding is indicated by dashed lines.

## supplementary crystallographic information

### Comment

The pyrazoline derivatives are well known nitrogen-containing heterocyclic compounds which show various biological activities and pharmacological properties (Palaska *et al.*, 1996). Some of them can be anti-bacterial and anti-fungal, others are anti-diabetic, anti-inflammatory and also active against many Mycobacterias (Ahn *et al.*, 2004; Yar *et al.*, 2006). As the stereochemistry may be an important modulator of biological activity, the crystal structure of the title compound has been determined.

The molecular structure is shown in Fig. 1. There are two chlorophenyl rings bonded with two pyrazoline rings in *cis*-arrangement, and these two pyrazoline rings are further bonded with the same pyridine ring. The central pyridine ring and two attached pyrazoline rings are nearly coplanar with the dihedral angles of 1.32 (2) and 4.88 (2)°, whereas the dihedral angles between each chlorophenyl plane and the attached pyrazoline planes are 86.78 (1) and 77.70 (1)°.

In the crystal structure, there are weak intermolecular C—H···O hydrogen bonding (Table 1 and Fig. 2).

### Experimental

2,6-Dimethyl-3,5-di-[3-(4-chloro-phenyl)-acryloyl-pyridine (1 mmol, 0.436 g) and 85% hydrazine hydrate solution (4 mmol, 0.235 g) were dissolved in 5 ml of acetic acid solution. The solution was refluxed for 8 h, and allowed to cool to room temperature. The reaction mixture was poured into crushed ice, then neutralized with dilute sodium hydroxide solution. The solid separated was filtered off, washed with water, dried and recrystallized from ethyl acetate to give a colorless compound in a yield of of 40%. Single crystals suitable for X-ray analysis were obtained form tetrahydrofuran at room temperature.

### Refinement

All H atoms were placed in calculated positions, with C—H = 0.93–0.99 Å, and included in the final cycles of refinement using a riding model, U_iso_(H) = 1.5U_eq_(C) (for methyl groups) or 1.2U_eq_(C) (for others). There is a void of 56 Å^3^ in the crystal structure, but no solvent molecule could be located reasonably.

### Crystal data

**Table:**

C_29_H_27_Cl_2_N_5_O_2_	*F*_000_ = 572
*M**_r_* = 548.46	*D*_x_ = 1.268 Mg m^−^^3^
Monoclinic, *P*2_1_	Melting point = 547–549 K
Hall symbol: P 2yb	Mo *K*α radiation λ = 0.71073 Å
*a* = 12.345 (3) Å	Cell parameters from 2029 reflections
*b* = 9.6763 (19) Å	θ = 2.7–20.6º
*c* = 13.268 (3) Å	µ = 0.26 mm^−^^1^
β = 115.00 (3)º	*T* = 296 (2) K
*V* = 1436.4 (5) Å^3^	Block, colorless
*Z* = 2	0.30 × 0.20 × 0.20 mm

### Data collection

**Table:**

Bruker SMART CCD area-detector diffractometer	3736 independent reflections
Radiation source: fine-focus sealed tube	2520 reflections with *I* > 2σ(*I*)
Monochromator: graphite	*R*_int_ = 0.027
*T* = 173(2) K	θ_max_ = 25.0º
ω scans	θ_min_ = 2.7º
Absorption correction: multi-scan(SADABS; Sheldrick, 1996)	*h* = −14→14
*T*_min_ = 0.926, *T*_max_ = 0.950	*k* = −5→11
7509 measured reflections	*l* = −15→15

### Refinement

**Table:**

Refinement on *F*^2^	Hydrogen site location: inferred from neighbouring sites
Least-squares matrix: full	H-atom parameters constrained
*R*[*F*^2^ > 2σ(*F*^2^)] = 0.036	*w* = 1/[σ^2^(*F*_o_^2^) + (0.0287*P*)^2^] where *P* = (*F*_o_^2^ + 2*F*_c_^2^)/3
*wR*(*F*^2^) = 0.077	(Δ/σ)_max_ < 0.001
*S* = 0.91	Δρ_max_ = 0.14 e Å^−^^3^
3736 reflections	Δρ_min_ = −0.15 e Å^−^^3^
344 parameters	Extinction correction: none
1 restraint	Absolute structure: Flack (1983), 1031 Friedel pairs
Primary atom site location: structure-invariant direct methods	Flack parameter: 0.06 (6)
Secondary atom site location: difference Fourier map	

### Special details

**Table:**

Geometry. All e.s.d.'s (except the e.s.d. in the dihedral angle between two l.s. planes) are estimated using the full covariance matrix. The cell e.s.d.'s are taken into account individually in the estimation of e.s.d.'s in distances, angles and torsion angles; correlations between e.s.d.'s in cell parameters are only used when they are defined by crystal symmetry. An approximate (isotropic) treatment of cell e.s.d.'s is used for estimating e.s.d.'s involving l.s. planes.
Refinement. Refinement of *F*^2^ against ALL reflections. The weighted *R*-factor *wR* and goodness of fit *S* are based on *F*^2^, conventional *R*-factors *R* are based on *F*, with *F* set to zero for negative *F*^2^. The threshold expression of *F*^2^ > σ(*F*^2^) is used only for calculating *R*-factors(gt) *etc*. and is not relevant to the choice of reflections for refinement. *R*-factors based on *F*^2^ are statistically about twice as large as those based on *F*, and *R*- factors based on ALL data will be even larger.

### Fractional atomic coordinates and isotropic or equivalent isotropic displacement parameters (Å^2^)

**Table:**

	*x*	*y*	*z*	*U*_iso_*/*U*_eq_	
Cl1	0.82566 (10)	0.19017 (14)	0.84787 (9)	0.1067 (4)	
Cl2	0.93108 (9)	−0.23669 (14)	0.26803 (10)	0.1163 (4)	
N4	0.3040 (2)	0.0470 (3)	−0.03488 (19)	0.0535 (7)	
O2	0.4646 (2)	0.0216 (3)	−0.19685 (16)	0.0699 (7)	
N2	0.2426 (2)	0.4329 (3)	0.4064 (2)	0.0591 (7)	
C3	0.3001 (3)	0.2513 (3)	0.1935 (2)	0.0518 (8)	
H3A	0.3772	0.2901	0.2141	0.062*	
C2	0.2559 (3)	0.1586 (3)	0.1053 (2)	0.0472 (8)	
N5	0.3995 (2)	0.0474 (3)	−0.06575 (19)	0.0520 (7)	
N1	0.0772 (2)	0.1372 (3)	0.1322 (2)	0.0595 (7)	
C8	0.2922 (3)	0.3870 (3)	0.3447 (2)	0.0511 (8)	
N3	0.3251 (2)	0.5240 (3)	0.4848 (2)	0.0588 (7)	
C21	0.5046 (3)	0.1276 (3)	0.0106 (2)	0.0498 (8)	
H21A	0.5260	0.1990	−0.0324	0.060*	
C1	0.1420 (3)	0.1018 (3)	0.0771 (2)	0.0533 (8)	
C4	0.2348 (3)	0.2885 (3)	0.2519 (2)	0.0501 (8)	
C19	0.3333 (3)	0.1291 (3)	0.0493 (2)	0.0474 (8)	
C20	0.4534 (2)	0.1978 (3)	0.0844 (2)	0.0503 (8)	
H20A	0.5048	0.1814	0.1641	0.060*	
H20B	0.4447	0.2986	0.0705	0.060*	
C13	0.5391 (3)	0.4494 (3)	0.5838 (3)	0.0547 (8)	
C24	0.6109 (2)	0.0369 (3)	0.0729 (2)	0.0475 (8)	
O1	0.3715 (2)	0.6813 (3)	0.6201 (2)	0.0844 (8)	
C7	0.0821 (3)	−0.0002 (4)	−0.0160 (3)	0.0701 (10)	
H7A	0.0072	−0.0321	−0.0150	0.105*	
H7B	0.1351	−0.0793	−0.0061	0.105*	
H7C	0.0650	0.0446	−0.0874	0.105*	
C29	0.6086 (3)	−0.0608 (4)	0.1481 (3)	0.0580 (9)	
H29A	0.5379	−0.0706	0.1593	0.070*	
C11	0.2948 (4)	0.6055 (4)	0.5529 (3)	0.0677 (10)	
C9	0.4157 (3)	0.4450 (3)	0.3751 (2)	0.0564 (9)	
H9A	0.4160	0.5088	0.3169	0.068*	
H9B	0.4743	0.3702	0.3860	0.068*	
C18	0.5107 (3)	0.3598 (4)	0.6500 (3)	0.0598 (9)	
H18A	0.4298	0.3507	0.6388	0.072*	
C10	0.4435 (3)	0.5226 (4)	0.4848 (2)	0.0578 (9)	
H10A	0.4694	0.6193	0.4797	0.069*	
C25	0.7154 (3)	0.0477 (4)	0.0600 (3)	0.0642 (10)	
H25A	0.7208	0.1143	0.0097	0.077*	
C17	0.5992 (3)	0.2823 (4)	0.7333 (3)	0.0667 (9)	
H17A	0.5787	0.2220	0.7790	0.080*	
C22	0.3836 (3)	0.0014 (3)	−0.1681 (3)	0.0563 (9)	
C28	0.7057 (3)	−0.1451 (4)	0.2079 (3)	0.0667 (10)	
H28A	0.7016	−0.2112	0.2591	0.080*	
C14	0.6579 (3)	0.4611 (4)	0.6040 (3)	0.0742 (11)	
H14A	0.6801	0.5241	0.5611	0.089*	
C27	0.8074 (3)	−0.1310 (4)	0.1916 (3)	0.0684 (10)	
C6	0.0396 (3)	0.2566 (5)	0.2730 (3)	0.0917 (14)	
H6A	−0.0334	0.2013	0.2372	0.138*	
H6B	0.0187	0.3549	0.2660	0.138*	
H6C	0.0800	0.2318	0.3519	0.138*	
C5	0.1217 (3)	0.2283 (3)	0.2175 (3)	0.0572 (9)	
C16	0.7161 (3)	0.2940 (4)	0.7486 (3)	0.0690 (10)	
C26	0.8133 (3)	−0.0363 (5)	0.1185 (3)	0.0753 (11)	
H26A	0.8842	−0.0276	0.1074	0.090*	
C15	0.7466 (3)	0.3822 (5)	0.6863 (3)	0.0814 (12)	
H15A	0.8277	0.3908	0.6980	0.098*	
C12	0.1685 (3)	0.5944 (5)	0.5398 (3)	0.0878 (12)	
H12A	0.1567	0.6570	0.5924	0.132*	
H12B	0.1525	0.4992	0.5548	0.132*	
H12C	0.1136	0.6197	0.4637	0.132*	
C23	0.2705 (3)	−0.0739 (4)	−0.2390 (3)	0.0778 (11)	
H23A	0.2731	−0.1018	−0.3089	0.117*	
H23B	0.2019	−0.0129	−0.2548	0.117*	
H23C	0.2629	−0.1561	−0.1994	0.117*	

### Atomic displacement parameters (Å^2^)

**Table:**

	*U*^11^	*U*^22^	*U*^33^	*U*^12^	*U*^13^	*U*^23^
Cl1	0.0941 (8)	0.1130 (9)	0.1006 (8)	0.0245 (7)	0.0290 (6)	0.0137 (7)
Cl2	0.0693 (7)	0.1272 (11)	0.1354 (10)	0.0249 (7)	0.0267 (6)	0.0115 (8)
N4	0.0520 (16)	0.065 (2)	0.0518 (16)	−0.0009 (14)	0.0295 (13)	−0.0093 (15)
O2	0.0865 (17)	0.0762 (18)	0.0682 (15)	−0.0036 (13)	0.0531 (14)	−0.0109 (13)
N2	0.0685 (18)	0.064 (2)	0.0524 (16)	0.0096 (16)	0.0333 (15)	−0.0045 (15)
C3	0.0518 (18)	0.058 (2)	0.0517 (18)	−0.0006 (17)	0.0278 (16)	−0.0058 (17)
C2	0.0498 (18)	0.051 (2)	0.0464 (18)	0.0020 (15)	0.0262 (16)	−0.0037 (16)
N5	0.0536 (16)	0.0614 (19)	0.0479 (15)	−0.0026 (14)	0.0282 (13)	−0.0081 (14)
N1	0.0504 (16)	0.070 (2)	0.0659 (18)	−0.0028 (14)	0.0323 (15)	−0.0081 (16)
C8	0.063 (2)	0.051 (2)	0.0437 (18)	0.0075 (17)	0.0274 (17)	−0.0005 (16)
N3	0.0655 (18)	0.062 (2)	0.0556 (16)	0.0036 (15)	0.0319 (15)	−0.0152 (15)
C21	0.0572 (19)	0.050 (2)	0.0508 (18)	−0.0131 (17)	0.0315 (16)	−0.0076 (17)
C1	0.0519 (19)	0.059 (2)	0.0488 (19)	−0.0016 (17)	0.0213 (16)	−0.0033 (17)
C4	0.060 (2)	0.050 (2)	0.0499 (19)	0.0034 (17)	0.0320 (16)	0.0011 (17)
C19	0.0526 (19)	0.047 (2)	0.0435 (18)	0.0018 (16)	0.0214 (15)	−0.0054 (16)
C20	0.0570 (19)	0.051 (2)	0.0503 (18)	−0.0052 (16)	0.0297 (16)	−0.0076 (17)
C13	0.067 (2)	0.051 (2)	0.054 (2)	−0.0064 (18)	0.0341 (18)	−0.0127 (17)
C24	0.0502 (19)	0.049 (2)	0.0514 (19)	−0.0069 (17)	0.0292 (16)	−0.0083 (17)
O1	0.109 (2)	0.0778 (19)	0.0720 (17)	0.0029 (17)	0.0441 (16)	−0.0235 (16)
C7	0.065 (2)	0.077 (3)	0.076 (2)	−0.013 (2)	0.037 (2)	−0.018 (2)
C29	0.052 (2)	0.063 (2)	0.067 (2)	−0.0037 (18)	0.0330 (18)	−0.006 (2)
C11	0.084 (3)	0.066 (3)	0.057 (2)	0.021 (2)	0.034 (2)	−0.002 (2)
C9	0.075 (2)	0.052 (2)	0.052 (2)	−0.0078 (18)	0.0363 (18)	−0.0114 (17)
C18	0.064 (2)	0.066 (3)	0.061 (2)	−0.0023 (19)	0.0382 (19)	−0.0068 (19)
C10	0.071 (2)	0.055 (2)	0.056 (2)	−0.0092 (19)	0.0355 (19)	−0.0090 (18)
C25	0.066 (2)	0.070 (3)	0.072 (2)	−0.008 (2)	0.043 (2)	−0.001 (2)
C17	0.085 (3)	0.066 (3)	0.066 (2)	0.004 (2)	0.047 (2)	0.001 (2)
C22	0.067 (2)	0.054 (2)	0.052 (2)	0.0047 (18)	0.0293 (19)	−0.0077 (17)
C28	0.070 (2)	0.069 (3)	0.071 (2)	−0.002 (2)	0.039 (2)	0.002 (2)
C14	0.079 (3)	0.078 (3)	0.080 (3)	−0.020 (2)	0.047 (2)	−0.001 (2)
C27	0.051 (2)	0.076 (3)	0.073 (2)	0.003 (2)	0.0206 (19)	−0.009 (2)
C6	0.075 (2)	0.126 (4)	0.101 (3)	−0.013 (3)	0.063 (2)	−0.034 (3)
C5	0.053 (2)	0.068 (3)	0.059 (2)	0.0039 (18)	0.0314 (17)	−0.0082 (19)
C16	0.069 (2)	0.072 (3)	0.062 (2)	0.003 (2)	0.025 (2)	−0.006 (2)
C26	0.054 (2)	0.092 (3)	0.092 (3)	−0.007 (2)	0.042 (2)	−0.010 (2)
C15	0.064 (3)	0.090 (3)	0.095 (3)	−0.007 (2)	0.039 (2)	0.005 (3)
C12	0.087 (3)	0.101 (3)	0.082 (3)	0.027 (2)	0.042 (2)	−0.015 (2)
C23	0.076 (2)	0.088 (3)	0.069 (2)	−0.005 (2)	0.030 (2)	−0.032 (2)

### Geometric parameters (Å, °)

**Table:**

Cl1—C16	1.752 (4)	C7—H7B	0.9800
Cl2—C27	1.760 (4)	C7—H7C	0.9800
N4—C19	1.291 (3)	C29—C28	1.389 (4)
N4—N5	1.401 (3)	C29—H29A	0.9500
O2—C22	1.227 (3)	C11—C12	1.498 (5)
N2—C8	1.291 (3)	C9—C10	1.543 (4)
N2—N3	1.415 (3)	C9—H9A	0.9900
C3—C4	1.382 (4)	C9—H9B	0.9900
C3—C2	1.390 (4)	C18—C17	1.399 (4)
C3—H3A	0.9500	C18—H18A	0.9500
C2—C1	1.406 (4)	C10—H10A	1.0000
C2—C19	1.465 (4)	C25—C26	1.390 (5)
N5—C22	1.362 (4)	C25—H25A	0.9500
N5—C21	1.484 (3)	C17—C16	1.374 (4)
N1—C1	1.336 (3)	C17—H17A	0.9500
N1—C5	1.355 (4)	C22—C23	1.502 (4)
C8—C4	1.480 (4)	C28—C27	1.367 (4)
C8—C9	1.511 (4)	C28—H28A	0.9500
N3—C11	1.365 (4)	C14—C15	1.400 (5)
N3—C10	1.460 (4)	C14—H14A	0.9500
C21—C24	1.502 (4)	C27—C26	1.359 (5)
C21—C20	1.530 (4)	C6—C5	1.509 (4)
C21—H21A	1.0000	C6—H6A	0.9800
C1—C7	1.508 (4)	C6—H6B	0.9800
C4—C5	1.399 (4)	C6—H6C	0.9800
C19—C20	1.508 (4)	C16—C15	1.348 (5)
C20—H20A	0.9900	C26—H26A	0.9500
C20—H20B	0.9900	C15—H15A	0.9500
C13—C18	1.381 (4)	C12—H12A	0.9800
C13—C14	1.380 (4)	C12—H12B	0.9800
C13—C10	1.519 (4)	C12—H12C	0.9800
C24—C25	1.376 (4)	C23—H23A	0.9800
C24—C29	1.384 (4)	C23—H23B	0.9800
O1—C11	1.231 (4)	C23—H23C	0.9800
C7—H7A	0.9800		
			
C19—N4—N5	106.8 (2)	C8—C9—H9B	111.1
C8—N2—N3	107.0 (3)	C10—C9—H9B	111.1
C4—C3—C2	121.8 (3)	H9A—C9—H9B	109.1
C4—C3—H3A	119.1	C13—C18—C17	121.0 (3)
C2—C3—H3A	119.1	C13—C18—H18A	119.5
C3—C2—C1	117.9 (2)	C17—C18—H18A	119.5
C3—C2—C19	116.4 (3)	N3—C10—C13	114.6 (3)
C1—C2—C19	125.6 (3)	N3—C10—C9	100.4 (2)
C22—N5—N4	120.4 (3)	C13—C10—C9	111.7 (3)
C22—N5—C21	124.5 (3)	N3—C10—H10A	109.9
N4—N5—C21	113.8 (2)	C13—C10—H10A	109.9
C1—N1—C5	119.9 (3)	C9—C10—H10A	109.9
N2—C8—C4	124.1 (3)	C24—C25—C26	121.8 (3)
N2—C8—C9	113.5 (3)	C24—C25—H25A	119.1
C4—C8—C9	122.4 (3)	C26—C25—H25A	119.1
C11—N3—N2	122.1 (3)	C16—C17—C18	119.6 (3)
C11—N3—C10	124.0 (3)	C16—C17—H17A	120.2
N2—N3—C10	113.8 (2)	C18—C17—H17A	120.2
N5—C21—C24	112.2 (2)	O2—C22—N5	117.7 (3)
N5—C21—C20	101.0 (2)	O2—C22—C23	123.1 (3)
C24—C21—C20	114.1 (2)	N5—C22—C23	119.1 (3)
N5—C21—H21A	109.7	C27—C28—C29	118.7 (3)
C24—C21—H21A	109.7	C27—C28—H28A	120.7
C20—C21—H21A	109.7	C29—C28—H28A	120.7
N1—C1—C2	121.2 (3)	C13—C14—C15	121.6 (3)
N1—C1—C7	114.6 (3)	C13—C14—H14A	119.2
C2—C1—C7	124.2 (3)	C15—C14—H14A	119.2
C3—C4—C5	116.5 (3)	C26—C27—C28	120.7 (3)
C3—C4—C8	116.6 (3)	C26—C27—Cl2	120.5 (3)
C5—C4—C8	126.9 (3)	C28—C27—Cl2	118.8 (3)
N4—C19—C2	123.7 (3)	C5—C6—H6A	109.5
N4—C19—C20	114.7 (2)	C5—C6—H6B	109.5
C2—C19—C20	121.6 (3)	H6A—C6—H6B	109.5
C19—C20—C21	103.0 (2)	C5—C6—H6C	109.5
C19—C20—H20A	111.2	H6A—C6—H6C	109.5
C21—C20—H20A	111.2	H6B—C6—H6C	109.5
C19—C20—H20B	111.2	N1—C5—C4	122.7 (3)
C21—C20—H20B	111.2	N1—C5—C6	114.0 (3)
H20A—C20—H20B	109.1	C4—C5—C6	123.3 (3)
C18—C13—C14	117.5 (3)	C15—C16—C17	120.6 (3)
C18—C13—C10	121.9 (3)	C15—C16—Cl1	119.8 (3)
C14—C13—C10	120.3 (3)	C17—C16—Cl1	119.5 (3)
C25—C24—C29	116.6 (3)	C27—C26—C25	119.8 (3)
C25—C24—C21	122.3 (3)	C27—C26—H26A	120.1
C29—C24—C21	121.1 (3)	C25—C26—H26A	120.1
C1—C7—H7A	109.5	C16—C15—C14	119.6 (3)
C1—C7—H7B	109.5	C16—C15—H15A	120.2
H7A—C7—H7B	109.5	C14—C15—H15A	120.2
C1—C7—H7C	109.5	C11—C12—H12A	109.5
H7A—C7—H7C	109.5	C11—C12—H12B	109.5
H7B—C7—H7C	109.5	H12A—C12—H12B	109.5
C28—C29—C24	122.5 (3)	C11—C12—H12C	109.5
C28—C29—H29A	118.7	H12A—C12—H12C	109.5
C24—C29—H29A	118.7	H12B—C12—H12C	109.5
O1—C11—N3	118.8 (3)	C22—C23—H23A	109.5
O1—C11—C12	124.8 (3)	C22—C23—H23B	109.5
N3—C11—C12	116.4 (3)	H23A—C23—H23B	109.5
C8—C9—C10	103.2 (2)	C22—C23—H23C	109.5
C8—C9—H9A	111.1	H23A—C23—H23C	109.5
C10—C9—H9A	111.1	H23B—C23—H23C	109.5
			
C4—C3—C2—C1	−0.5 (5)	N2—N3—C11—C12	−0.4 (5)
C4—C3—C2—C19	179.3 (3)	C10—N3—C11—C12	179.1 (3)
C19—N4—N5—C22	−162.3 (3)	N2—C8—C9—C10	8.9 (3)
C19—N4—N5—C21	5.3 (3)	C4—C8—C9—C10	−171.8 (3)
N3—N2—C8—C4	−179.9 (3)	C14—C13—C18—C17	−1.0 (5)
N3—N2—C8—C9	−0.6 (3)	C10—C13—C18—C17	173.1 (3)
C8—N2—N3—C11	170.7 (3)	C11—N3—C10—C13	74.2 (4)
C8—N2—N3—C10	−8.9 (3)	N2—N3—C10—C13	−106.2 (3)
C22—N5—C21—C24	−79.9 (4)	C11—N3—C10—C9	−165.9 (3)
N4—N5—C21—C24	113.0 (3)	N2—N3—C10—C9	13.7 (3)
C22—N5—C21—C20	158.2 (3)	C18—C13—C10—N3	17.2 (4)
N4—N5—C21—C20	−8.9 (3)	C14—C13—C10—N3	−168.8 (3)
C5—N1—C1—C2	−0.6 (5)	C18—C13—C10—C9	−96.1 (3)
C5—N1—C1—C7	−179.9 (3)	C14—C13—C10—C9	77.8 (4)
C3—C2—C1—N1	1.0 (4)	C8—C9—C10—N3	−12.5 (3)
C19—C2—C1—N1	−178.8 (3)	C8—C9—C10—C13	109.4 (3)
C3—C2—C1—C7	−179.9 (3)	C29—C24—C25—C26	0.7 (5)
C19—C2—C1—C7	0.4 (5)	C21—C24—C25—C26	179.7 (3)
C2—C3—C4—C5	−0.2 (5)	C13—C18—C17—C16	−0.9 (5)
C2—C3—C4—C8	179.4 (3)	N4—N5—C22—O2	170.1 (3)
N2—C8—C4—C3	179.2 (3)	C21—N5—C22—O2	3.8 (5)
C9—C8—C4—C3	0.0 (4)	N4—N5—C22—C23	−11.4 (4)
N2—C8—C4—C5	−1.2 (5)	C21—N5—C22—C23	−177.6 (3)
C9—C8—C4—C5	179.6 (3)	C24—C29—C28—C27	−0.1 (5)
N5—N4—C19—C2	179.1 (3)	C18—C13—C14—C15	1.9 (5)
N5—N4—C19—C20	1.1 (3)	C10—C13—C14—C15	−172.3 (3)
C3—C2—C19—N4	−178.9 (3)	C29—C28—C27—C26	0.1 (5)
C1—C2—C19—N4	0.9 (5)	C29—C28—C27—Cl2	179.5 (3)
C3—C2—C19—C20	−1.1 (4)	C1—N1—C5—C4	−0.2 (5)
C1—C2—C19—C20	178.7 (3)	C1—N1—C5—C6	−179.0 (3)
N4—C19—C20—C21	−6.5 (3)	C3—C4—C5—N1	0.6 (5)
C2—C19—C20—C21	175.5 (3)	C8—C4—C5—N1	−179.0 (3)
N5—C21—C20—C19	8.4 (3)	C3—C4—C5—C6	179.3 (3)
C24—C21—C20—C19	−112.2 (3)	C8—C4—C5—C6	−0.3 (5)
N5—C21—C24—C25	116.5 (3)	C18—C17—C16—C15	1.9 (5)
C20—C21—C24—C25	−129.5 (3)	C18—C17—C16—Cl1	−176.6 (3)
N5—C21—C24—C29	−64.7 (4)	C28—C27—C26—C25	0.3 (6)
C20—C21—C24—C29	49.4 (4)	Cl2—C27—C26—C25	−179.1 (3)
C25—C24—C29—C28	−0.3 (5)	C24—C25—C26—C27	−0.8 (5)
C21—C24—C29—C28	−179.3 (3)	C17—C16—C15—C14	−1.0 (6)
N2—N3—C11—O1	179.2 (3)	Cl1—C16—C15—C14	177.5 (3)
C10—N3—C11—O1	−1.3 (5)	C13—C14—C15—C16	−1.0 (6)

### Hydrogen-bond geometry (Å, °)

**Table:**

*D*—H···*A*	*D*—H	H···*A*	*D*···*A*	*D*—H···*A*
C9—H9A···O2^i^	0.99	2.59	3.358 (3)	135
C17—H17A···O2^ii^	0.95	2.50	3.359 (4)	151

Symmetry codes: (i) −*x*+1, *y*+1/2, −*z*; (ii) *x*, *y*, *z*+1.
